# Associations Between Levels of High-Sensitivity C-Reactive Protein and Outcome After Intracerebral Hemorrhage

**DOI:** 10.3389/fneur.2020.535068

**Published:** 2020-10-06

**Authors:** Jing Wang, Wenjuan Wang, Yanfang Liu, Xingquan Zhao

**Affiliations:** ^1^Department of Neurology, Beijing Tiantan Hospital, Capital Medical University, Beijing, China; ^2^China National Clinical Research Center for Neurological Diseases, Beijing, China; ^3^Center of Stroke, Beijing Institute for Brain Disorders, Beijing, China; ^4^Beijing Key Laboratory of Translational Medicine for Cerebrovascular Disease, Beijing, China; ^5^Beijing Key Laboratory of Central Nervous System Injury, Beijing, China

**Keywords:** spot sign, hematoma expansion, spontaneous intracerebral hemorrhage, clinical outcome, high-sensitivity CRP

## Abstract

**Background:** Patients with spontaneous intracerebral hemorrhage (ICH) have high mortality and morbidity rates; approximately one-third of patients with ICH experience hematoma expansion (HE). The spot sign is an established and validated imaging marker for HE. High-sensitivity C-reactive protein (hs-CRP) is an established laboratory marker for inflammation and secondary brain injury following ICH.

**Objective:** To determine the association between the spot sign and hs-CRP, hematoma expansion, and clinical outcomes.

**Methods:** Between December 2014 and September 2016, we prospectively recruited 1,964 patients with acute symptomatic ICH at 13 hospitals in Beijing, China. Next, we selected 92 patients within 24 h of the onset of symptoms from this cohort for the present study. ICH was diagnosed in the emergency room by non-contrast computed tomography (NCCT) scans. Follow-up scans were carried out within 48 h to evaluate patients for HE. Multidetector computed tomography angiography (MDCTA) was also used to identify spot signs. Blood samples were collected from each patient at admission in EDTA tubes (for plasma) or vacutainer tubes (for serum). hs-CRP values were determined by a particle-enhanced immunoturbidimetric assay in the laboratory at Beijing Tiantan Hospital, Capital Medical University. Patients were categorized into two groups according to their hs-CRP levels (hs-CRP <3 mg/L, hs-CRP ≥3 mg/L).

**Results:** The incidences of spot sign and HE in our study cohort were 31.5 and 29.3%, respectively. Following the removal of potential confounding variables, stepwise-forward logistic regression analysis identified that an hs-CRP level ≥3 mg/L was not a significant indicator for either spot sign (*p* = 0.68) or HE (*p* = 0.07). However, an hs-CRP level ≥3 mg/L (odds ratio: 16.64, 95% confidence interval: 2.11–131.45, *p* = 0.008) was identified as an independent predictor of an unfavorable outcome 1 year after acute ICH.

**Conclusions:** Our analyses identified that an hs-CRP level ≥3 mg/L was a significant indicator for an unfavorable outcome 1 year after acute ICH.

## Introduction

Spontaneous intracerebral hemorrhage (ICH) is a devastating subtype of stroke with high global rates of mortality and morbidity (1). ICH is responsible for over half of all deaths caused by stroke, and functional independence after ICH is known to range from 12 to 39% 1 year or longer (1). Up to one-third of patients with ICH may experience hematoma expansion (HE), thus leading to a higher mortality rate and unfavorable clinical outcomes (2, 3). Therefore, it is vital that we develop methods to prevent and manage HE in patients experiencing acute ICH. The spot sign is a well-established and validated imaging marker for HE (4). However, identifying the spot sign requires the application of computed tomography angiography (CTA) with the additional injection of contrast materials and exposure to radiation. Furthermore, neuroradiologists are needed for processing and reconstructing images acquired by CTA. Previous randomized controlled trials reported that <25% of patients with ICH underwent CTA during the acute phase (5, 6). Other research studies have reported that a number of indicators are associated with HE and poor outcomes in patients with ICH, including non-contrast CT (NCCT) markers, such as computed tomography (CT) hypodensities (7), the black hole sign (8), the blend sign (9), and the island sign (10). However, there is an urgent need for further research to identify the prognostic value of biomarkers for HE.

Previous studies have shown that an acute inflammatory reaction may occur within minutes of an ICH, thus triggering an inflammatory cascade that exacerbates secondary brain injury (11). Levels of high-sensitivity C-reactive protein (hs-CRP), as an indicator of inflammation, tend to be higher following stroke. Studies have demonstrated that levels of hs-CRP are also associated with hematoma volume and clinical outcomes after ICH (12). However, the precise relationships between hs-CRP levels, the presence of a spot sign, and HE following spontaneous ICH have yet to be elucidated.

This study aimed to investigate the relationships between hs-CRP levels, the presence of a spot sign, HE, and poor clinical outcomes in a cohort of patients suffering from acute ICH.

## Materials and Methods

### Study Population

This study was based on data obtained from a cohort study conducted at 13 hospitals in Beijing, China. The cohort study protocol was approved by the Institutional Review Board (IRB) of Beijing Tiantan Hospital, Capital Medical University, Beijing, China. The ethics committee approved consent by proxy in the ethics statement. Additional ethics approval was required in some of the participating hospitals, and additional consent was obtained if required by the ethics board of specific sites. All patients who had the capacity to understand and sign the documentation provided to them, or their legally authorized representative, were required to provide written and informed consent in a manner that depended on the requirements of the ethics committee at each specific study site. Participating centers collected data and submitted it online to the coordinating center at Beijing Tiantan Hospital, Capital Medical University.

**Figure 1:**
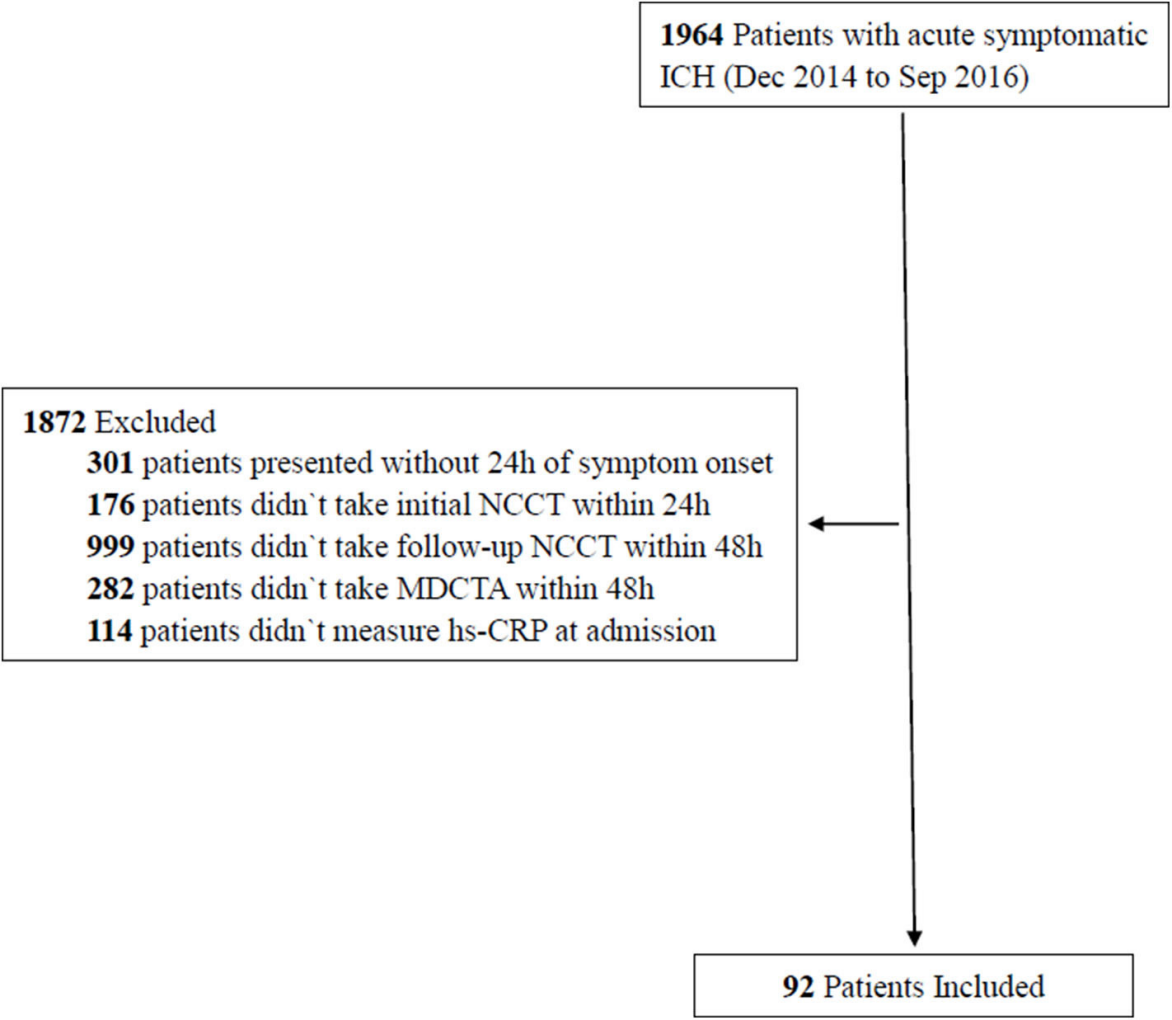
Flow chart of study patients.

Between December 2014 and September 2016, we recruited 1,964 patients, aged ≥18 years, who had been diagnosed with acute symptomatic ICH by computed tomography. Next, we selected 92 patients from this cohort for the present study (Figure 1). Our inclusion criteria were as follows: patients who presented within 24 h of the onset of symptoms, data relating to the initial NCCT were available, patients who underwent follow-up NCCT at 48 h and multidetector CT angiography (MDCTA) within 48 h, and hs-CRP data were collected at admission. Patients were excluded if they had a form of secondary ICH (hemorrhages resulting from aneurysm, vascular malformation, hemorrhagic infarction, tumors, or impaired coagulation), a history of acute or chronic infection, or underwent surgery prior to the follow-up NCCT and MDCTA.

### Collection of Clinical Information

Demographic and baseline characteristics were collected at admission, including age, gender, alcohol and tobacco use, history of hypertension/diabetes/stroke, concomitant medications (such as antihypertensive/antiplatelet medications), systolic blood pressure (SBP), and routine laboratory data (leukocyte count, platelet count, blood glucose, fibrinogen, activated partial thromboplastin time, and international normalized ratio). We also determined scores for the Glasgow Coma Scale (GCS) and the National Institutes of Health Stroke Scale (NIHSS) at the time of admission. At hospital discharge, antiplatelet medication, antihypertensive medication, in-hospital infections (lung/urinary tract), necessity of operation, and rehabilitation information were collected.

### Patient Follow-Up and Clinical Outcomes After ICH

One year after the indexed ICH events, we carried out telephone interviews to all of the patients to acquire a range of follow-up data related to functional outcomes, including the modified Rankin Scale (mRS) and the non-fatal recurrence of stroke. Weekly phone calls were carried out until three missed calls were recorded; at this point, patients were classified as being lost to follow-up. A structured interview protocol was used in all telephone follow-ups, and all interviews were carried out by trained personnel. An unfavorable clinical outcome was defined as an mRS score >2 after 1 year of follow-up (13).

### Radiological Information

The CT scan protocol was consistent with previous studies (14). In brief, an initial NCCT was performed, using standard clinical parameters (with an axial section thickness of 5 mm), on each patient who presented within 24 h of the onset of symptoms. A follow-up NCCT scan was also performed within 48 h of admission. A MDCTA scan was also performed to identify spot signs. MDCTA scans were performed with a bolus-tracking method by injecting 90 ml of non-ionic iodinated contrast (OPTIRAY 350) at a rate of 5 ml/s. Data arising from the MDCTA scan were then processed by a CT technologist. All images were viewed in a double-blinded manner on PACS workstations by one neuroradiologist and one neurologist who had no access to clinical data. These members of clinical staff were asked to identify spot signs in accordance with previously published criteria (15) and to exclude cases involving secondary ICH, such as those caused by aneurysms, vascular malformation, or venous sinus thrombosis. The two clinical staff had to agree on each case; differences in opinion were settled by consensus. We also recorded the time from symptom onset to initial NCCT, the location of the hematoma (supratentorial deep gray matter vs. other locations, such as lobar and infratentorial locations), and the presence of intraventricular hemorrhage (IVH). ICH volumes were determined by manually drawing a hematoma outline on the Siemens multidetector-row scanner using specialized software. HE was diagnosed when there was an increase in hematoma volume by >33% or >12.5 ml (16).

### Measurement and Classification of High-Sensitivity CRP

Blood samples were collected from each patient at admission in EDTA tubes (for plasma) or vacutainer tubes (for serum). Levels of hs-CRP were determined by a particle-enhanced immunoturbidimetric assay (Ultrasensitive CRP kit; Orion Diagnostica, Espoo, Finland) and an auto-analyzer (Hitachi 911; Hitachi, Tokyo, Japan) in the laboratory at Beijing Tiantan Hospital. Levels of hs-CRP level were categorized into two groups according to the laboratory cut-off value: hs-CRP <3 mg/L and hs-CRP ≥3 mg/L.

### Statistical Analysis

Statistical analyses were performed using the SPSS statistical package version 23.0 (SPSS Inc., Chicago, USA). Continuous variables are described as means ± standard deviation (SD) or as medians (with interquartile range, IQR) as appropriate. These data were compared using Student *t*-tests or Wilcoxon rank-sum tests, accordingly. Categorical variables are presented as numbers (percentages) and were compared using chi-squared tests. Multivariable logistic regression models were carried out using the stepwise-forward method (Wald) and used to calculate odds ratios (ORs) for the relationship between hs-CRP and the risks of spot sign, HE, and unfavorable outcomes 1 year after acute ICH. Variables that were known to be associated with spot sign, HE, or clinical outcomes 1 year after ICH were then included in a multivariate model. Stepwise regression used the SPSS default *p*-value of 0.05. Multicollinearity analysis indicated that the variance inflation factor (VIF) was <2.0 in the parsimonious model. All statistical analyses were two-tailed, and a *p*-value of 0.05 was considered to be statistically significant.

## Results

### Baseline Characteristics

We recruited 92 patients in this study; 54 of these patients had an hs-CRP level ≥3 mg/L. The baseline characteristics for all patients and groups according to hs-CRP levels are shown in Table 1. The median GCS score for the entire cohort was 14.0 (11.3, 15.0), and the mean NIHSS score was 11.8 ± 8.0. The mean duration of time between the onset of symptoms and the initial NCCT was 4.1 h, whereas the median hematoma volumes at baseline and follow-up were 22.4 ml (10.0, 48.4 ml) and 25.9 ml (11.0, 57.0 ml), respectively. Most of the hematoma was located in the supratentorial deep gray matter (54 patients, 58.7% of the cohort), followed by lobar locations (35 patients, 38.0% of the cohort), and infratentorial locations (2 patients, 2.2% of the cohort). Spot signs and HE were observed in 29 patients (31.5% of the cohort) and 27 patients (29.3% of the cohort), respectively. Thirty-three patients (35.9% of the cohort) had poor clinical outcomes 1 year after ICH. Patients with higher hs-CRP levels at admission were more likely to be male and have a larger hematoma volume at baseline and follow-up; these patients also tended to be more prone to infections and surgical interventions. These patients were also more likely to suffer from HE and poor outcomes 1 year after ICH. Five patients died within 1 year of ICH; all five of these patients had hs-CRP levels ≥3 mg/L.

**Table 1:** Baseline characteristics of all patients and patients grouped by hs-CRP levels.

		**hs-CRP**		
	**All (n = 92)**	** <3 mg/L (n = 38)**	**≥3 mg/L (n = 54)**	***p*-value**
Male gender	64 (69.6)	21 (55.3)	43 (79.6)	0.01
Age, year	53.0 (42.5, 60.0)	51.5 (41.8, 57.3)	56.0 (45.5, 62.0)	0.16
History of hypertension	58 (63.0)	21 (55.3)	37 (68.5)	0.16
History of diabetes	8 (8.7)	3 (7.9)	5 (9.3)	0.82
History of stroke	8 (8.7)	4 (10.5)	4 (7.4)	0.61
Current smoking	36 (39.1)	13 (34.2)	23 (42.6)	0.42
Alcohol	46 (50.0)	18 (47.4)	28 (51.9)	0.65
Antiplatelet medication	7 (7.6)	2 (5.3)	5 (9.3)	0.92
Antihypertensive medication	23 (25.0)	10 (26.3)	13 (24.1)	0.70
Infections (lung/urinary tract)	29 (31.5)	4 (10.5)	25 (46.3)	<0.01
Necessity of operation	25 (27.2)	3 (7.9)	22 (40.7)	<0.01
Rehabilitation	58 (63.0)	23 (60.5)	35 (64.8)	0.68
GCS score	14.0 (11.3, 15.0)	14.0 (12.8, 15.0)	13.5 (10.0, 15.0)	0.13
NIHSS score	11.8 ± 8.0	9.9 ± 7.2	13.2 ± 8.3	0.05
SBP, mmHg	163.5 ± 27.0	161.7 ± 28.7	164.8 ± 25.9	0.60
Time from onset to initial NCCT (h)	4.1 (2.3, 7.6)	4.6 (2.2, 7.2)	3.9 (2.4, 7.9)	0.90
Interval between initial NCCT and follow-up NCCT (h)	18.5 (12.0, 24.0)	19.0 (12.0, 25.0)	18.0 (13.0, 23.0)	0.58
Baseline hematoma volume (ml)	22.4 (10.0, 48.4)	14.5 (7.9, 27.1)	37.3 (14.9, 55.4)	<0.01
Hematoma volume at follow-up (ml)	25.9 (11.0, 57.0)	14.4 (8.0, 28.5)	39.8 (18.8, 67.4)	<0.01
Hematoma location				0.43
Lobar	35 (38.0)	15 (39.5)	20 (37.0)	
Deep localization	54 (58.7)	23 (60.5)	31 (57.4)	
Infratentorial location	2 (2.2)	0	2 (3.7)	
IVH	1 (1.1)	0	1 (1.9)	
Spot sign	29 (31.5)	12 (31.6)	17 (31.5)	0.99
Hematoma expansion	27 (29.3)	7 (18.4)	20 (37.0)	0.05
mRS 3–6 at 3 months	43 (46.7)	8 (21.1)	35 (64.8)	<0.01
mRS 3–6 at 1 year	33 (35.9)	3 (7.9)	30 (55.6)	<0.01
Non-fatal recurrence of stroke at 1 year	4 (4.3)	2 (5.3)	2 (3.7)	0.80

*Data are expressed as n (%), mean ± standard deviation (SD), or median (IQR), as appropriate*.

*GCS, Glasgow Coma Scale; hs-CRP, high-sensitivity C-reactive protein; IVH, intraventricular hemorrhage; mRS, modified Rankin Scale; NCCT, non-contrast CT; NIHSS, National Institutes of Health Stroke Scale; SBP, systolic blood pressure*.

### Spot Signs, Radiological Characteristics, and Marker Levels

Spot signs were detected in 29 out of the 92 recruited patients (31.5%). Patients with spot signs were more likely to have a larger hematoma volume at baseline and follow-up (baseline: 44.1 [18.8, 55.2 ml] vs. 16.2 ml [8.1, 38.2 ml], *p* = 0.01; follow-up: 44.4 [23.2, 67.2 ml] vs. 21.0 ml [8.7, 43.9 ml], *p* = 0.02). However, spot signs were more likely to occur in lobar locations, but less likely to occur in deep locations. Levels of hs-CRP were not significantly associated with spot signs (Table 2).

**Table 2:** Radiological characteristics and marker levels according to spot sign.

**Radiological data**	**No spot sign**	**Spot sign**	***p*-value**
	**(*n* = 63)**	**(*n* = 29)**	
Hematoma location			0.04
Lobar	20 (31.7)	15 (51.7)	
Deep localization	40 (63.5)	14 (48.3)	
IVH	2 (3.2)	0	
Infratentorial location	1 (1.6)	0	
Baseline hematoma volume (ml)	16.2 (8.1, 38.2)	44.1 (18.8, 55.2)	0.01
Hematoma volume at follow-up (ml)	21.0 (8.7, 43.9)	44.4 (23.2, 67.2)	0.02
Hematoma expansion	20 (31.7)	7 (24.1)	0.46
Time from onset to initial NCCT (h)	4.0 (2.3, 6.4)	4.4 (2.3, 9.4)	0.59
hs-CRP, mg/L	6.0 (1.8, 11.3)	4.0 (1.3, 11.9)	0.91

*Data are expressed as n (%), mean ± standard deviation (SD), or median (IQR), as appropriate*.

*hs-CRP, high-sensitivity C-reactive protein; IVH, intraventricular hemorrhage; NCCT, non-contrast CT*.

### Correlations Between hs-CRP, Spot Signs, and Hematoma Extension

Several variables were applied in the stepwise-forward logistic regression for spot signs, including gender, age, baseline hematoma volume, hematoma location, GCS score, NIHSS score, time from onset to initial NCCT, SBP, antiplatelet medication, and hs-CRP level. These analyses showed that only hematoma location created a good model. There were no useful indicators identified when all the other variables, as well as the incidence of spot signs, were included in stepwise-forward logistic regression for HE. In other words, an hs-CRP level ≥3 mg/L was not a significant indicator for either spot signs (*p* = 0.68) or HE (*p* = 0.07). However, hematoma location (OR: 3.08, 95% confidence interval [CI]: 1.31–7.22, *p* = 0.01) was identified as an independent predictor for spot signs.

### Associations Between hs-CRP Levels and Clinical Outcome

Next, all variables, including gender, sex, baseline hematoma volume, hematoma location, time from onset to initial NCCT, GCS scores, NIHSS scores at baseline, spot signs, HE, SBP, and hs-CRP levels, were applied into a stepwise-forward logistic regression for the clinical outcome 1 year after ICH. The best models included an hs-CRP level ≥3 mg/L, age, baseline hematoma volume, NIHSS score, and HE. Using these four selected indices, we then carried out multicollinearity analysis; the VIF of each of these parameters was <2.0. Thus, an hs-CRP level ≥3 mg/L (OR: 37.16, 95% CI: 2.78–496.46, *p* = 0.006), age (OR: 1.25, 95% CI: 1.08–1.45, *p* = 0.003), baseline hematoma volume (OR: 1.07, 95% CI: 1.01–1.13, *p* = 0.03), and HE (OR: 18.12, 95% CI: 1.41–233.32, *p* = 0.03) were identified as significant and independent predictors for an unfavorable outcome 1 year after acute ICH (Table 3).

**Table 3:** Stepwise-forward (Wald) logistic regression for 1-year clinical outcomes stratified by hs-CRP.

	**Logistic**		**Collinearity**
	**regression**		**statistics**
	**OR (95% CI)**	***p*-value**	**VIF**
hs-CRP (≥3 mg/L)	37.16 (2.78, 496.46)	0.006	1.23
Age	1.25 (1.08, 1.45)	0.003	1.05
Baseline hematoma volume, ml	1.07 (1.01, 1.13)	0.03	1.16
NIHSS score	1.14 (0.99, 1.31)	0.07	1.10
HE	18.12 (1.41, 233.32)	0.03	1.11

*HE, hematoma enlargement; hs-CRP, high-sensitivity C-reactive protein; NIHSS, National Institutes of Health Stroke Scale; VIF, variance inflation factor*.

*Data were adjusted for age, gender, baseline hematoma volume, hematoma location, time from onset to initial NCCT, GCS score, NIHSS score at baseline, spot sign, SBP, HE, and hs-CRP*.

## Discussion

In this study, we found that an hs-CRP level ≥3 mg/L was a significant indicator of unfavorable outcomes 1 year after ICH, but not an independent risk factor for either spot signs or HE.

Several other factors are worth noting. First, it has been shown that hematoma volume at admission is associated with poor clinical outcomes (17). In the present study, the stepwise-forward logistic regression for clinical outcome 1 year after acute ICH also showed that baseline hematoma volume was an independent predictor of unfavorable outcome 1 year after ICH. We also found that the baseline hematoma volume in patients with an hs-CRP level ≥3 mg/L (median: 37.3 ml) was over 2-fold higher than those in patients with an hs-CRP level <3 mg/L (median: 14.5 ml); the same trend was also evident for hematoma volume at follow-up (median: 39.8 vs. 14.4 ml). Thus, we conclude that hs-CRP is a good indicator for hematoma volume. We also found that hs-CRP level remained as an independent predictor for poor clinical outcome even after the ICH volume and HE were controlled in logistic regression, thus suggesting that hs-CRP may also represent a useful marker for further secondary injuries caused by ICH.

Previous research has reported that elevated hs-CRP levels are associated with poor outcomes in several cardiovascular diseases, including ischemic stroke (18, 19), subarachnoid hemorrhage (20, 21), and acute coronary artery diseases (22). Our present study showed that elevated hs-CRP levels were correlated with poor outcomes in patients suffering from ICH. All of these poor outcomes may arise from the acute inflammatory response that can occur several minutes after ICH and then trigger systemic inflammation (11, 23). hs-CRP is an established laboratory marker for inflammation and poor clinical outcomes after ICH (24). However, whether hs-CRP is related to spot signs or HE has not been ascertained in previous research. Our present study showed that elevated hs-CRP levels were associated with poor clinical outcomes, as reported by previous studies, but were not associated with spot signs or HE. The mechanisms underlying the association between hs-CRP levels and poor clinical outcomes have not been fully elucidated, although an inflammatory cascade is believed to play an important role in this process. hs-CRP may cause direct disruption of the blood–brain barrier (BBB) and induce endothelial dysfunction (25); collectively, these processes may result in secondary brain injury, thus leading to poor clinical outcomes (12) and HE. Previous research demonstrated that the peak hs-CRP concentration occurs mainly within 48 h of acute ICH, and that the hs-CRP level at 24 h is a more accurate predictor of poor outcomes than at admission (26). In our present study, we only collected blood samples at admission; it is possible that this practice may have interfered with our investigations of the association between hs-CRP, spot signs, and HE.

Previous research showed that the spot sign is an independent risk factor for HE and poor clinical outcomes (27–30), with a sensitivity of 62% and a specificity of 88% (30). Previous studies also reported that the incidence of spot sign ranged from 19 to 46%, despite different time intervals between the onset of symptoms and initial imaging (15, 29, 31). In the present study, we detected the spot sign in 31.5% of our patient cohort; this incidence was similar to that reported previously. It is thought that the spot sign represents the site of vessel rupture and active bleeding during the early stages, but represents the site of physiological hemostasis, associated microaneurysms, or aneurysm-like lesions during subsequent stages (32, 33). It is also evident that “spot sign” and “contrast extravasation” are often misunderstood and considered to be the same. The “spot sign” refers to enhanced foci ≥1 mm in size within a hematoma on CTA images, whereas “contrast extravasation” refers to an increase in contrast density on enhanced CT images (33). Therefore, we used the term “spot sign” in our current study as our analysis included CTA images. A number of risk factors have been associated with spot signs, including anticoagulation, the apolipoprotein Eε2 allele, a larger initial hematoma volume, early presentation, lower GCS scores at admission, a mean arterial blood pressure >120 mmHg, and the presence of IVH (34). HE often occurs within 4 h of the onset of symptoms, although in some cases, HE occurs within the subsequent 21 h (35). The risk factors for HE are similar to those for spot signs (35). At present, there are a number of different criteria used to diagnose HE; in the present study, we used the most widely accepted criteria (35). We found that HE is a risk factor for unfavorable outcomes 1 year after ICH; spot sign was not a risk factor for an unfavorable outcome. Overall, our data suggest that elevated hs-CRP levels in the early stages of ICH may be associated with poor outcomes. Our findings may have some potential clinical applications. For example, future clinical trials designed to test hemostatic agents and neuroprotective drugs should include hs-CRP levels as an efficient biomarker. Large-scale studies are now needed to verify our current findings.

This study has several limitations that need to be considered. First, the data used in our study were obtained from an existing database and not collected prospectively from consecutive patients. This practice may have resulted in potential bias, such as selection bias, as patients with higher NIHSS scores and larger hematomas may not have been able to undergo follow-up CT scans within 48 h. Thus, the presence of spot signs and HE could have been underestimated. Second, almost one-third of the patients in our study underwent neurosurgery 48 h after the onset of symptoms; this may have influenced our analysis of clinical outcomes 1 year after ICH. However, the Surgical Trial in Intracerebral Hemorrhage (STICH) trial (36, 37) and the STICH II trial (38) showed that there were no significant differences in functional outcomes between a surgery group and a medical group of patients with spontaneous supratentorial lobar intracerebral hematomas. Moreover, the minimally invasive surgery with thrombolysis in intracerebral hemorrhage evacuation (MISTIE III) trial showed that the minimally invasive surgical evacuation of ICH did not improve the clinical outcomes 1 year after ICH when compared with non-surgical medical care (37). Further studies are now needed to verify these findings. Third, we recruited patients who presented within 24 h of the onset of symptoms; MDCTA was usually performed within 48 h and reported in a previous study (26); this practice could have also led to the underestimation of HE. Nevertheless, the incidence of HE in our study was similar to that reported in other studies. Finally, our study cohort only included 92 patients and may, therefore, have had limited statistical power. Further research now needs to investigate the prognostic value of biomarkers for spot signs and HE.

## Conclusion

In conclusion, our study demonstrated that an hs-CRP level ≥3 mg/L was a significant indicator of unfavorable clinical outcomes 1 year after acute ICH. However, in this study, we only selected 92 patients from a cohort of 1,872 patients, which may lead to selection bias with <5% of patients. Further prospective studies, with large sample sizes, are now needed to identify the mechanisms underlying this relationship.

## Data Availability Statement

The datasets generated for this study are available on request to the corresponding author.

## Ethics Statement

The studies involving human participants were reviewed and approved by the Institutional Review Board (IRB) of the Beijing Tiantan Hospital, Capital Medical University. The patients/participants provided their written informed consent to participate in this study.

## Author Contributions

JW conducted the statistical analyses and drafted the original manuscript. WW interpreted the data and conceptualized the study. YL performed the imaging assessment. XZ researched the literature and designed the study.

## Conflict of Interest

The authors declare that the research was conducted in the absence of any commercial or financial relationships that could be construed as a potential conflict of interest.
